# Membrane Cholesterol Is a Critical Determinant for Hippocampal Neuronal Polarity

**DOI:** 10.3389/fnmol.2021.746211

**Published:** 2021-10-21

**Authors:** Mini Jose, Aiswarya Sivanand, Chaitra Channakeshava

**Affiliations:** Centre for Neuroscience, Indian institute of Science, Bangalore, India

**Keywords:** hippocampal neuronal development, membrane cholesterol, axo-dendritic specification, lipid homeostasis, neurite outgrowth, neuronal polarity, cholesterol labeling

## Abstract

Maintaining a normal cholesterol balance is crucial for the functioning of a healthy brain. Dysregulation in cholesterol metabolism and homeostasis in the brain have been correlated to various neurological disorders. The majority of previous studies in primary cultures focus on the role of cholesterol balance in neuronal development after polarity has been established. Here we have investigated how transient alteration of membrane lipids, specifically cholesterol, affects neuronal development and polarity in developing hippocampal neurons prior to polarity establishment, soon after initiation of neurite outgrowth. We observed that temporary cholesterol perturbation affects axonal and dendritic development differentially in an opposing manner. Transient membrane cholesterol deficiency increased neuronal population with a single neurite, simultaneously generating a second population of neurons with supernumerary axons. Brief replenishment of cholesterol immediately after cholesterol sequestering rescued neuronal development defects and restored polarity. The results showed a small window of cholesterol concentration to be complementing neurite outgrowth, polarity reestablishment, and in determining the normal neuronal morphology, emphasizing the critical role of precise membrane lipid balance in defining the neuronal architecture. Membrane cholesterol enhancement modified neurite outgrowth but did not significantly alter polarity. Cholesterol sequestering at later stages of development has shown to enhance neurite outgrowth, whereas distinct effects for neurite development and polarity were observed at early developmental stages, signifying the relevance of precise membrane cholesterol balance in altering neuronal physiology. Our results confirm cholesterol to be a key determinant for axo-dendritic specification and neuronal architecture and emphasize the possibility to reverse neuronal developmental defects caused by cholesterol deficiency by modulating membrane cholesterol during the early developmental stages.

## Introduction

The axo-dendritic specification is a defining feature for the unique architecture of neurons. A well-coordinated orchestra of positive and negative feedback signals is thought to regulate the breaking of symmetry and establishment of neuronal polarity with a singular axon and multiple dendrites in neurons (Arimura and Kaibuchi, 2007; Barnes and Polleux, 2009; Schelski and Bradke, 2017; Takano et al., 2019). The functional distinction of the different neuronal compartments including the axons and the dendrites is brought about by the selective filtering of molecules giving a distinct molecular composition, allowing a unidirectional flow of signals during synaptic transmission (Song et al., 2009). Different functional and molecular determinants for this specification have been characterized over the years (Leterrier et al., 2015; Gumy et al., 2017; Leterrier, 2018). Membrane lipids including sphingolipids and cholesterol have been shown to have a prominent role in the selective localization of molecules particularly in the axolemma by lipid-protein interactions (Ledesma et al., 1998). Several evidences emphasize the relevance of lipid homeostasis, particularly that of lipid rafts and cholesterol, in various physiological and pathological processes (Vance, 2012; Sviridov et al., 2020; Vona et al., 2021). Cholesterol metabolism and homeostasis are thought to have critical roles in maintaining a healthy brain (Zhang and Liu, 2015). Dysregulation in the lipid balance has been correlated to diverse neurodegenerative conditions including Alzheimer’s disease, Parkinson’s disease, Huntington’s disease, Niemann Pick type C disease, and recently to cancer (Vance, 2012; Wang and Song, 2012; Leoni and Caccia, 2015; Hussain et al., 2019; Vona et al., 2021). Proteins such as amyloid precursor protein (APP) vital in the pathological outcome of neurological disorders such as AD have been recently shown to be critically involved in the cholesterol turnover for neuronal activity, supporting the importance of cholesterol homeostasis in a wider context (Pierrot et al., 2013; Grimm et al., 2017; Cho et al., 2020).

Autonomous cholesterol synthesis has been shown to occur in neuronal soma but not in axons (Vance et al., 1994). The majority of cholesterol synthesis in the brain occurs in glial cells, particularly during active myelination covering the axons, and significantly reduces in the mature brain once the myelination is complete (Quan et al., 2003; Dietschy and Turley, 2004; Funfschilling et al., 2012). Therefore, cholesterol is thought to have a prominent role in regulating neuronal development and neurite outgrowth. Cholesterol mediated neurite outgrowth has been reported to be dependent on the brain regions, being different between the cortical and hippocampal regions (Ko et al., 2005). Cholesterol modulation is also thought to regulate dendritic outgrowth, with little effect on the axonal extension (Fan et al., 2002). Most of these studies have been conducted on later developmental stages of neurons (stages 3–5), when the neuronal polarity establishment and axon determination have already been initiated (Dotti et al., 1988). Though the lipid balance and compositionality of the membrane are thought to be critical in neurite outgrowth, the fine regulation of membrane cholesterol on the axonal and dendritic development particularly at very early stages, from the initiation of protrusions to breaking of symmetry and polarity establishment (stages 1–3), has never been explored in detail. In this study, we have addressed the role of membrane lipid regulation, particularly cholesterol, in regulating neurite outgrowth and development and in determining the axo-dendritic specification at very early stages of neurite growth.

We transiently sequestered cholesterol to different levels in rat hippocampal neurons at DIV1 (days *in vitro*) for varying durations. The alterations in membrane cholesterol levels after transient sequestering were verified using an unesterified cholesterol assay. The neurons were allowed to grow to DIV3 and imaged using axonal and dendritic markers. Interestingly, we observed opposing effects for cholesterol sequestering on the axonal and dendritic growth and a marked shift in neuronal polarity. Along with an increase in the neuronal population with a single neurite, cholesterol sequestering also generated a second population of neurons with supernumerary axons. The neuro-morphological and polarity defects were rescued upon briefly replenishing the neurons with exogenous cholesterol, confirming this effect to be cholesterol dependent. The short concentration window of optimal cholesterol replenishment revealed the critical nature of membrane lipid balance on maintaining normal neuronal morphology. Conversely, transient cholesterol enhancement complemented neurite extension but did not alter polarity. Increased duration of cholesterol sequestering elevated the population of cells entering apoptosis. Our results elucidate that membrane cholesterol is a critical determinant for axonal and dendritic outgrowth, and for regulating axo-dendritic specification and neuronal architecture in rat hippocampal neurons.

## Materials and Methods

### Cell Culture

Primary cultures were prepared from P0/P1 rat pups (Sprague-Dawley) as described earlier (Goslin and Banker, 1989). The hippocampus was dissected out from the rodent brain in Hibernate Media (Thermofisher Scientific). The dissected hippocampi were trypsinized (0.25% Trypsin), washed 3×, and dissociated into single cells by syringing using needles of 2 pore sizes sequentially to remove all neurites (21 G and 25 G, BD Precision Glide Needle). The cells were plated on 18 mm diameter thickness corrected coverslips (Marienfeld Superior) coated with Poly-L-Lysine (1 mg/ml, Sigma Aldrich) after resuspending in Neurobasal media (Thermofisher Scientific) complemented with B27, Glutamate, and Normocin (Invivogen; referred to as Complete Neurobasal media) at a density of 50 cells/mm^2^.

### Drug Treatment and Immunocytochemistry

The cells were fixed at DIV1, DIV2, and DIV3 with 4% PFA+sucrose and labeled with antibodies against Map2 (Merck Millipore #AB5622, rabbit) and Tau (Merck Millipore #05–348, mouse). Alexa 488 and Alexa 647 secondary antibodies (Thermofisher Scientific) marked Map2 and Tau, respectively for dual color labeling.

For cholesterol sequestration, the cells were treated transiently with differing concentrations (2.5 mM, 5 mM, 7.5 mM, and 10 mM) of Methyl β cyclodextrin (MβCD, Sigma Aldrich C4555) for 10 min or 20 min at 37 deg at DIV1. The MβCD stock (100 mM) was reconstituted in water and the working concentrations were prepared in Complete Neurobasal media for treatment. After treatment, the cells were washed thrice in prewarmed Complete Neurobasal media and allowed to grow to DIV3 before fixing them. For cholesterol replenishment, the cells treated with 5 mM MβCD for 10 min were washed and further treated for 10 min with differing concentrations of exogenous cholesterol (Sigma Aldrich C4951, Cl:MβCD, 0.5 mM, 1.0 mM, 1.5 mM, and 2.0 mM) at 37 deg. The Cholesterol stock (100 mM) was reconstituted in water and the working concentrations were prepared in Complete Neurobasal media for treatment. The cells were again washed thrice in prewarmed Complete Neurobasal media and allowed to grow to DIV3 before fixing them. The treated cells were labeled with antibodies against Map2 and Tau marked by Alexa 488 and Alexa 647, respectively. The coverslips were mounted using Prolong antifade agent (Thermofisher Scientific).

### Cholesterol Labeling and Viability Assay

For measuring unesterified cholesterol, the control and cells treated with varying concentrations of MβCD (2.5 mM, 5 mM, 7.5 mM, and 10 mM) for 10 min and 20 min were labeled at DIV3 using a membrane marker for lipid rafts tagged with Alexa 555 (Thermofisher Scientific V34404). Membrane labeling was done in Complete Neurobasal media for 10 min at 4 deg. The cells were then washed, fixed with 4% PFA+sucrose and labeled with Filipin III (Sigma Aldrich F4767) at a concentration of 50 μg/ml in PBS for 2 h at RT and overnight at 4 deg, after which they were washed and imaged in PBS.

The membrane damage to the cells induced on cholesterol sequestering for varying durations was assessed using a Live/Dead Cell imaging kit (Thermofisher Scientific R37601) which uses Calcein AM cell permeable dye and Bobo-3 Iodide as live and dead cell indicators, respectively. The live control and treated cells (10 mM MβCD, 10 min, 20 min, and 30 min) were labeled at DIV3 for 15 min at RT and imaged in an extracellular imaging buffer. For quantifying the viability of cells undergoing cholesterol sequestering, the control and cells treated for varying durations (10 mM MβCD, 10 min, 20 min, and 30 min) were fixed at DIV3 and labeled with an antibody (Cleaved Caspase 3, rabbit, #9661, Cell Signaling Technology) which recognize endogenous levels of activated Caspase 3 marked by Alexa 555. The coverslips were mounted using Prolong antifade agent containing DAPI (Thermofisher Scientific).

### Microscope Image Acquisition and Quantitative Analysis

Images were acquired an inverted microscope Zeiss Axio Observer Z1 equipped with a 40× objective, halogen lamp and an ScMOS camera (pco.edge). Specific filters for Alexa 488 and Alexa 647 were used for acquisition. 3D stacks of total volume thickness of 3 μm with a plane thickness of 200 nm were acquired. The images were exported, and all processing and image analyses were performed using Metamorph 7.0. Maximum intensity projections of the images were generated. For analysis of neuronal development profile, length of the longest neurite, length of dendrites, number of neurites per cell and the neuronal polarity ratio were quantified. For specific sets of experiments, additional analysis included calculation of the percentage of cells with single neurite, percentage of neurons with multiple axons, and polarity ratio of neurons displaying multiple axons, as explained. Due to the minimal variability between neuronal cultures, Mean ± SEM is presented for all data. The statistical difference between the means was calculated by One-way Anova (Graph Pad Prism 9). ****p < 0.0001, ***p < 0.001, **p < 0.01, *p < 0.05 and ns non-significant.

Imaging of cells labeled with Filipin III was performed similarly using a 40× objective. Due to the high photobleaching of Filipin III, cells were focused using the lipid raft membrane marker tagged with Alexa 555 and 3D stacks of total volume thickness of 3 μm with a plane thickness of 200 nm were acquired for both channels. DsRed filter was used for Alexa 555 acquisition, whereas Filipin fluorescence was acquired using a filter combination 420–480 nm excitation and 500–550 nm emission. Quantification of Filipin fluorescence was done from maximum intensity projections of 3D stacks. The images were thresholded for automatic detection of light objects to form a mask. The regions mapping the cells were transferred to the original images to quantify the corresponding average intensity of the cells.

For quantifying the viability of cells undergoing cholesterol sequestering, images of control and treated cells were acquired using a 20× objective. For the live cell assay, images were acquired for green and orange channels using GFP and DsRed filters, respectively. The images were thresholded and assessed by the Count nuclei App in Metamorph software. The ratio of orange (dead) to green (live) cells in each field was quantified and normalized using the mean value of control cells. For the caspase 3 assay, images were acquired using DAPI and DsRed filters. The percentage of DAPI marked cells colabeled with Caspase 3 (positive, dead cells) vs. DAPI only cells (negative, live cells) was quantified for each field by a Cell Scoring App in Metamorph software. The ratio of the percentage of dead vs. live cells was assessed and normalized using the mean value of control cells.

The statistical difference between the means of each condition (fluorescence intensity/ratio) was calculated by One-way Anova (Graph Pad Prism 9). ****p < 0.0001, ***p < 0.001, **p < 0.01, *p < 0.05 and ns non-significant.

## Results

### Assessing Rat Hippocampal Neuronal Development Profile

**Figure 1:**
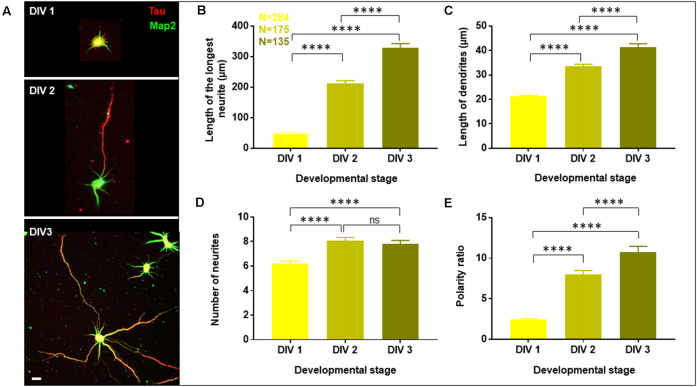
Developmental profile of optimized primary cultures of rat hippocampal neurons. Neurons were grown to three developmental stages namely DIV 1, 2, and 3, fixed and labeled with Tau (red) and Map2 (green) as axonal and dendritic markers, respectively. **(A)** A gallery of cells at DIV 1, 2, and 3 is presented. **(B–E)** Neuronal development was assessed by quantifying the length of the longest neurite **(B)**, length of dendrites **(C)**, number of neurites in each cell **(D)**, and the polarity ratio **(E)**, which was calculated from the ratio of the longest neurite to the average length of dendrites of each cell. N denotes the number of cells from four independent cultures. One-way Anova was performed to test the significance of the difference between the means. ****p < 0.0001, ns denotes non-significant. Scale bar indicates 20 μm.

To understand the role of cholesterol on neuronal development, it was critical to assess a homogeneous neuronal population at different stages of development. Cell culture protocols were optimized to prepare homogenous primary hippocampal neuronal cultures from postnatal day 0 or 1 (P0/P1) rat pups. The morphology of pyramidal neurons in these cultures was assessed at DIV1, DIV2, and DIV3 (Figure 1). The homogeneity of cultures was verified using Tau (axonal marker) and Map2 (somato-dendritic marker) antibodies, which showed >90% of the plated neurons to be undifferentiated at DIV1 vs. >90% differentiated at DIV3. A gallery of low magnification images of cells at each developmental stage is presented in Supplementary Figure 1. 3D image stacks of total volume thickness of 3 μm of Tau and Map2 labeled neurons at different stages were acquired at higher magnification (Figure 1). All quantifications were performed on the maximum intensity projections of the stacks with an overlay of both channels. The experimental and analytical procedures are outlined in Supplementary Figure 2.

Neuronal development and growth profile were analyzed by quantifying the length of the longest neurite (axon), length of the dendrites (all neurites except the longest) and the number of neurites per cell. This classification of neurites into axon and dendrites was entirely based on their length and not their specific labeling. Neuronal polarity was assessed based on the ratio of the length of the longest neurite (axon) to the average length of the dendrites, which is referred to as the neuronal polarity ratio. The higher values of the neuronal polarity ratio indicated the asymmetric pattern of the neurons with a single long axon and short dendrites, while small values of polarity ratio showed a symmetric growth pattern, where the neurites were similar in length. The conventional semi-automated software for neuro-morphology assessment including Simple Neurite Tracer (SNT), Neural Circuit Tracer (NCT), AutoNeuriteJ, etc. are limited by various factors such as lack of sensitivity to detect fine neuronal processes for precise characterization, exclusion of overlapping cells, the requirement of additional labeling and lack of distinction of axo-dendritic processes (Chothani et al., 2011; Longair et al., 2011; Boulan et al., 2020). Therefore, manual tracing of neurites was performed from the periphery of the soma to the distal end of the neurites. All projections and bifurcations including the secondary and tertiary processes were considered and added to the length of the primary process. Thresholding of neurite length was performed where a cut-off length of 10 μm was introduced to exclude filopodia. The quantification of all parameters including the length of the longest neurite, length of dendrites, number of neurites per cell, and polarity ratio was done using the filtered values (Supplementary Figure 2B).

A gallery of rat hippocampal neurons of different stages namely, DIV1, DIV2, and DIV3 is presented (Figure 1A). A steep increase in the length of the longest neurite (referred to as the axon) was observed from DIV1 (51.73 ± 1.86 μm) to DIV3 (328.2 ± 14.53 μm), with DIV2 (212.7 ± 8.38 μm) being intermediate (Figure 1B, Table 1). The dendrites (rest of the neurites except the longest) also displayed a gradual increase in their length along development, though not as fast as the axon (Figure 1C, Table 1). The length of the dendrites increased from 21.27 ± 0.3 μm at DIV1 to 41.42 ± 1.35 μm at DIV3. The number of neurites per cell showed a slight increase with the maturity of neurons from 6 ± 0.2 at DIV1 to 8 ± 0.3 at DIV 2 and DIV3 (Figure 1D, Table 1). Neuronal development involves both growth of the neuronal processes as well as an axo-dendritic specification or neuronal polarity establishment. Different technical challenges including lack of reliable markers at the early developmental stages made it hard to determine whether the specification between the two neuronal processes has been established. Therefore, the neuronal polarity ratio or the ratio of the length of the longest neurite (axon) to the average length of the dendrites was assessed, which showed the polarity status of the neuron. The polarity ratio displayed a drastic increase from 2.47 ± 0.09 at DIV1 to 10.72 ± 0.75 at DIV3 (Figure 1E, Table 1), confirming this to be a reliable parameter to assess the switch of a neuron from a symmetric to asymmetric growth pattern. Along with the qualitative assessment of polarity establishment using Tau and Map2 as axonal and dendritic markers, quantifying the polarity ratio allowed us to make a reliable and additional assessment on the multiple morphological phenotypes observed by membrane lipid perturbations.

**Table 1:** Developmental profile of primary cultures of rat hippocampal neurons.

Developmental stage	DIV1	DIV2	DIV3
Length of the longest neurite (μm)	51.73 ± 1.86	212.7 ± 8.38	328.2 ± 14.53
Length of dendrites (μm)	21.27 ± 0.30	33.49 ± 0.85	41.42 ± 1.35
Number of neurites per cell	6 ± 0.2	8 ± 0.3	8 ± 0.3
Polarity ratio	2.47 ± 0.09	7.99 ± 0.49	10.72 ± 0.75

*The table shows the morphological properties of primary cultures of rat hippocampal neurons at three developmental stages namely, DIV1, 2, and 3. Mean ± SEM is presented for N number of cells as shown in Figure 1 from four independent cultures*.

To account for the variability between cultures, all quantifications were done across the same cultures at different stages. The minimal error observed for the different parameters across the cultures showed the robustness of the data and purity of the cultures suggesting its homogenous nature, enabling us to quantify the reported parameters in a reliable manner. A significant increase in the length and number of neurites as well as in the percentage of polarized cells was observed during rat hippocampal neuronal development from DIV1 to DIV3.

### Cholesterol Sequestering Has Opposing Effects on Axon vs. Dendrites

Having a homogenous neuronal population, which could be developmentally assessed, allowed us to investigate the role of membrane cholesterol in two important aspects of neuronal development, in neurite outgrowth and polarity establishment. In contrast to the majority of studies which employed polarity established cells, the experiments described here were conducted at very early stages of development before the axodendritic specification was established (stages 1–3). Also, instead of chronic deprivation of cholesterol from the membrane surface, we adopted a minimally invasive approach where cholesterol was transiently sequestered for a few minutes. The treated cells were then washed and allowed to grow for 2 more days, after which they were fixed and immunolabeled with Tau and Map2 (Supplementary Figure 2Ai). This experimental paradigm allowed us to directly address the effects of a transient membrane lipid alteration on neuronal morphology and development.

**Figure 2:**
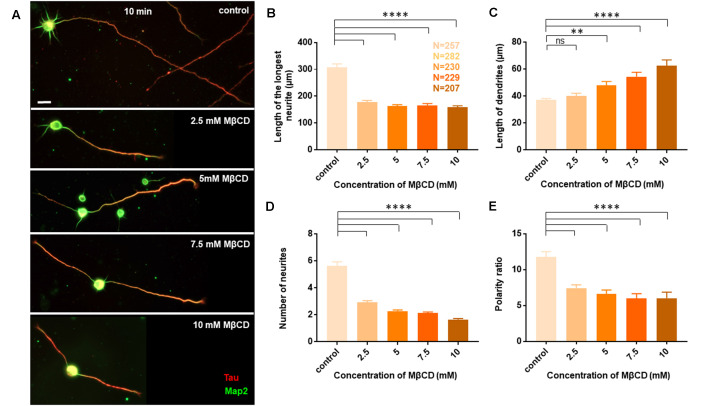
Neurons exhibit opposing effects for axonal and dendritic growth upon transient perturbation of membrane cholesterol. Neurons at DIV1 were transiently treated with varying concentrations of MβCD for 10 min to sequester cholesterol. The cells were labeled with Tau (red) and Map2 (green) and imaged at DIV3. **(A)** A gallery of control and cells treated with varying concentrations of MβCD for 10 min is presented. **(B–E)** Represents the length of the longest neurite **(B)**, length of dendrites **(C)**, number of neurites in each cell **(D)**, and the neuronal polarity ratio **(E)**. Cholesterol sequestered neurons displayed a significant defect in axonal growth but complemented dendritic growth. N denotes the number of cells from five independent cultures. One-way Anova was performed to test the significance of the difference between the means. ****p < 0.0001, **p < 0.01 and ns denotes non-significant. Scale bar indicates 20 μm.

Neurons at DIV1 were transiently treated with the cholesterol sequestering drug, Methyl β cyclodextrin (MβCD), at various concentrations (2.5 mM, 5 mM, 7.5 mM, and 10 mM) for 10 min and analyzed at DIV3 (Figure 2). Control cells were treated similarly with the highest amount of carrier alone (water). A gallery of cells including the control and those treated with varying concentrations of MβCD is presented (Figure 2A). We observed that transient cholesterol perturbation exerted effects on both neurite outgrowth and neuronal polarity establishment. This also revealed two distinct populations of neurons with different phenotypes on neuronal morphology.

**Figure 3:**
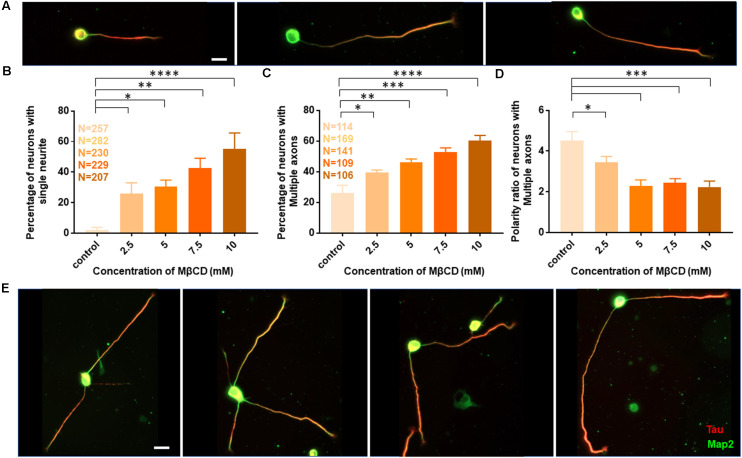
Transient cholesterol sequestering affects neuronal arborization and polarity. **(A)** A gallery of neurons treated with MβCD for 10 min, exhibiting single neurites, is presented. **(B)** Percentage of cells with single neurites upon application of varying concentrations of MβCD. **(C)** Percentage of cells with supernumerary axons upon application of varying concentrations of MβCD for 10 min.** (D)** Polarity ratio of neurons exhibiting supernumerary axons as calculated from **(C)**. **(E)** A gallery of treated neurons displaying supernumerary axons is presented. Cholesterol sequestered neurons treated with MβCD displayed single neurites and multiple axons, exhibiting a severe shift in neuronal polarity. The polarity ratio was significantly reduced in neurons exhibiting multiple axons, showing symmetric growth for the affected cells. N denotes the number of cells from five independent cultures for **(B)** and from three independent cultures for **(C,D)**. One-way Anova was performed to test the significance of the difference between the means. ****p < 0.0001, ***p < 0.001, **p < 0.01, *p < 0.05. Scale bar indicates 20 μm.

The length of the longest neurite (referred to as the axon) was significantly attenuated with the increasing concentration of MβCD. The length of the axon decreased from 307.2 ± 13.12 μm in control neurons to 158.7 ± 5.49 μm when MβCD concentration was increased to 10 mM (Figure 2B, Table 2). Interestingly, cholesterol perturbation by MβCD increased the length of the dendrites (Figure 2C, Table 2). In contrast to the control neurons where the average length of dendrites was 37.04 ± 1.13 μm, the dendritic length increased to 62.38 ± 4.39 μm upon augmenting the MβCD concentration to 10 mM. A stark effect on neuronal arborization was observed on transient cholesterol sequestration by MβCD. The neurite number per cell reduced from 6 ± 0.3 to 2 ± 0.1 with increasing MβCD concentration (Figure 2D, Table 2). The strong effect of cholesterol sequestering on neuronal arborization resulted in a second phenotype, i.e., the population of cells with a single neurite (Figure 3A). The percentage of cells with a single neurite increased drastically with cholesterol perturbation (Figure 3B). In control cultures, the percentage of neurons with a single neurite was negligible (2 ± 2%). In striking contrast, the MβCD treated cultures exhibited a significant increase in neurons with a single neurite from 26 ± 7% in 2.5 mM MβCD to 55 ± 10% in 10 mM MβCD (Figure 3B, Table 2).

**Table 2:** Developmental alterations of rat hippocampal neurons upon transient sequestering of cholesterol for 10 min.

Sample	Control	2.5 mM MβCD	5 mM MβCD	7.5 mM MβCD	10 mM MβCD
Length of the longest neurite (μm)	307.2 ± 13.12	178.0 ± 6.12	162.4 ± 5.66	165.9 ± 6.67	158.7 ± 5.49
Length of dendrites (μm)	37.04 ± 1.13	39.94 ± 2.08	47.82 ± 2.95	54.40 ± 3.26	62.38 ± 4.39
Number of neurites per cell	6 ± 0.3	3 ± 0.1	2 ± 0.1	2 ± 0.1	2 ± 0.1
Polarity ratio	11.79 ± 0.73	7.43 ± 0.46	6.62 ± 0.53	5.97 ± 0.68	6.02 ± 0.85
Percentage of cells with single neurite (%)	2 ± 2	26 ± 7	31 ± 4	43 ± 6	55 ± 10
Percentage of cells with multiple axon (%)	26 ± 5	39 ± 2	46 ± 2	53 ± 3	60 ± 3
Polarity ratio of cells with multiple axon	4.51 ± 0.44	3.45 ± 0.29	2.30 ± 0.29	2.43 ± 0.22	2.24 ± 0.29

*The table shows the neuro-morphological modifications upon transient cholesterol sequestering for 10 min using varying concentrations of MβCD. Mean ± SEM is presented for N number of cells as shown in Figures 2 and 3 from five and three independent cultures*.

Quantification of all growth parameters was done from the same cultures to minimize variability between cultures. The minimal error in data obtained from these cultures confirmed the robustness of the data. These results showed distinct effects of transient cholesterol perturbation on the growth of axon vs. dendrites, significantly affecting both neurite outgrowth and neuronal arborization.

### Cholesterol Sequestering Affects Neuronal Polarity

We next asked whether the membrane cholesterol perturbation affected neurite outgrowth alone or whether neuronal polarity establishment was also affected. The neuronal polarity ratio displayed a significant change from 11.79 ± 0.73 in control down to 5.97 ± 0.68 with cholesterol sequestering by MβCD (Figure 2E, Table 2), confirming that neuronal polarity was affected as well. As an independent assessment to dissect out the effect of cholesterol sequestration on neuronal polarity, we performed a qualitative comparison of cells immunolabeled for the axonal marker Tau and classified cells based on three criteria: (1) showing no polarity (undifferentiated), (2) polarized (with a single axon), and (3) polarized (with multiple or supernumerary axons).

Using Tau to label axons can be challenging at early developmental stages since Tau labels all neurites initially and then gets restricted to axons in mature neurons (Supplementary Figure 1). Since the developmental stage of interest in this study was very early when polarity is just getting established, we assessed the percentage of control neurons displaying multiple neurites with Tau labeling or with supernumerary axons (Table 2, Figures 3C,E). We observed that the percentage of cells displaying supernumerary axons significantly rose from 26 ± 5% to 60 ± 3% with increasing MβCD concentration (Table 2, Figure 3C). The multiple axons observed in cholesterol perturbed cells exhibited very strong and comparable levels of Tau labeling among them, whereas the majority of control cells displayed only a single neurite that was strongly labeled for Tau. For a better understanding of the effect of cholesterol sequestering on neuronal polarity, we compared the neuronal polarity ratio of the control cells and the MβCD treated cells displaying supernumerary axons. We observed that the polarity ratio of the MβCD treated cells with multiple axons significantly decreased (2.24 ± 0.29) compared to control cells displaying the same (4.51 ± 0.44, Table 2). Low values of the polarity ratio showed that the neurons exhibited a symmetric morphology following cholesterol sequestration compared to an asymmetric growth in control cells, which had a much longer axon compared to the dendrites. Therefore, in addition to a reduction in the length of the axon with increasing membrane cholesterol perturbation, there was also an increase in the percentage of neurons reversing to symmetric growth and displaying supernumerary axons. This unique population of cells with multiple axons would also explain the distinct effects observed previously, where there was an increase in the length of all the neurites except the axon upon cholesterol sequestration (Figure 2C). Therefore, the shift in polarity and an elevated population of neurons displaying supernumerary axons would largely account for the increase in length of dendrites observed with higher cholesterol sequestering.

### Neuronal Developmental Defects and Opposing Effects on Axon and Dendrites Is Independent of Duration of Cholesterol Perturbation

**Figure 4:**
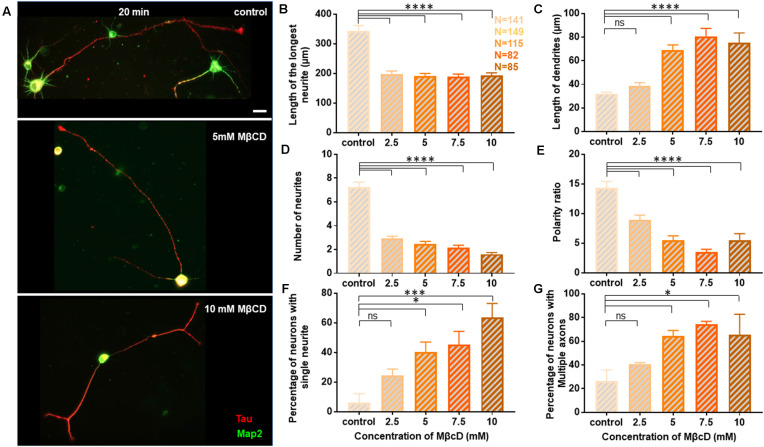
Neuronal developmental defects in growth and polarity are retained independent of the duration of transient cholesterol perturbation. Neurons at DIV1 were transiently treated with varying concentrations of MβCD for 20 min to sequester cholesterol. The cells were labeled with Tau (red) and Map2 (green) and imaged at DIV3. **(A)** A gallery of control and cells treated with varying concentrations of MβCD for 20 min is presented. **(B–E)** Represents the length of the longest neurite **(B)**, length of dendrites **(C)**, number of neurites in each cell **(D)**, and the polarity ratio **(E)** at varying concentrations of MβCD. **(F,G)** Represents the percentage of treated neurons displaying single neurites **(F)** and supernumerary axons **(G)**, respectively. Cholesterol sequestration for a longer duration resulted in neurons displaying significant defects in axonal growth but complementing dendritic growth, similar to shorter perturbation. Consistent with acute transient perturbation, longer cholesterol sequestration also resulted in neurons displaying single neurites and multiple axons, exhibiting a severe shift in neuronal polarity. N denotes the number of cells from three independent cultures. One-way Anova was performed to test the significance of the difference between the means. ****p < 0.0001, ***p < 0.001, *p < 0.05 and ns non-significant. Scale bar indicates 20 μm.

Since distinct effects of cholesterol perturbation were observed on neuronal growth and polarity, we next addressed whether these effects were dependent on the duration of perturbation. For this, rat hippocampal neurons at DIV1 were treated with varying concentrations of MβCD, but the duration of cholesterol sequestering was doubled to 20 min (Figure 4). The cells were washed and allowed to grow to DIV3 when they were immunolabeled with Tau and Map2. All quantifications were done as described previously for the 10 min perturbation. A gallery of cells treated with differing concentrations of MβCD from 2.5 mM, 5 mM, 7.5 mM to 10 mM for 20 min is presented along with control neurons treated with the carrier alone (Figure 4A). Similar to that reported for the 10 min treatment, the growth of the axon was strongly attenuated for the 20 min treatment as well, with increasing MβCD concentrations (Figure 4B). The length of the axon significantly reduced in MβCD treated neurons (193.7 ± 8.63 μm) when compared to the control cells (344 ± 17.74 μm; Figure 4B, Table 3). Membrane cholesterol perturbation for 20 min caused an increase in the dendritic length, as observed in 10 mM MβCD treated cells (75.18 ± 8.36 μm), compared to that in the control condition (32.08 ± 1.21 μm; Figure 4C, Table 3). Neuronal arborization was also severely affected with a longer duration of cholesterol perturbation, wherein the number of neurites per cell reduced from 7 ± 0.4 to 2 ± 0.1 at higher MβCD concentrations (Figure 4D, Table 3). Similar to that observed with 10 min treatment, cholesterol perturbation for 20 min also resulted in an increase in the population of neurons with a single neurite (6 ± 6% in control to 64 ± 9% in treated cells; Figure 4F, Table 3). Neurons also displayed a significant decrease in their polarity ratio on longer cholesterol sequestering (14.32 ± 1.13 in control cells; 5.49 ± 1.12 in treated cells; Figure 4E, Table 3). Cholesterol sequestering for 20 min also resulted in an increase in the number of cells displaying supernumerary axons (26 ± 9% in control; 66 ± 17% in treated cells; Figure 4G, Table 3). Together these observations suggest that the neuronal developmental defects observed upon cholesterol perturbation were not affected by the duration of perturbation. The effects observed after 10 min and 20 min treatment were similar and followed the same pattern. However, in contrast to membrane cholesterol perturbation for 10 min duration, which resulted in a gradual transition of morphological changes between the different concentrations, the effects observed with 20 min perturbation were abrupt and stronger.

**Table 3:** Developmental alterations of rat hippocampal neurons upon transient sequestering of cholesterol for 20 min.

Sample	Control	2.5 mM MβCD	5 mM MβCD	7.5 mM MβCD	10 mM MβCD
Length of the longest neurite (μm)	344 ± 17.74	198.1 ± 10.26	190.7 ± 9.08	189.1 ± 8.53	193.7 ± 8.63
Length of dendrites (μm)	32.08 ± 1.21	38.52 ± 2.92	68.98 ± 4.51	80.74 ± 6.61	75.18 ± 8.36
Number of neurites per cell	7 ± 0.4	3 ± 0.2	2 ± 0.2	2 ± 0.2	2 ± 0.1
Polarity ratio	14.32 ± 1.13	8.94 ± 0.81	5.48 ± 0.77	3.52 ± 0.45	5.49 ± 1.12
Percentage of cells with single neurite (%)	6 ± 6	24 ± 4	40 ± 6	45 ± 8	64 ± 9
Percentage of cells with multiple axon (%)	26 ± 9	41 ± 1	64 ± 5	74 ± 2	66 ± 17

*The table illustrates the alterations in morphological properties of rat hippocampal neurons upon transient cholesterol sequestering for 20 min using varying concentrations of the cholesterol sequestering drug MβCD. Mean ± SEM is presented for N number of cells as shown in Figure 4 from three independent cultures*.

**Figure 5:**
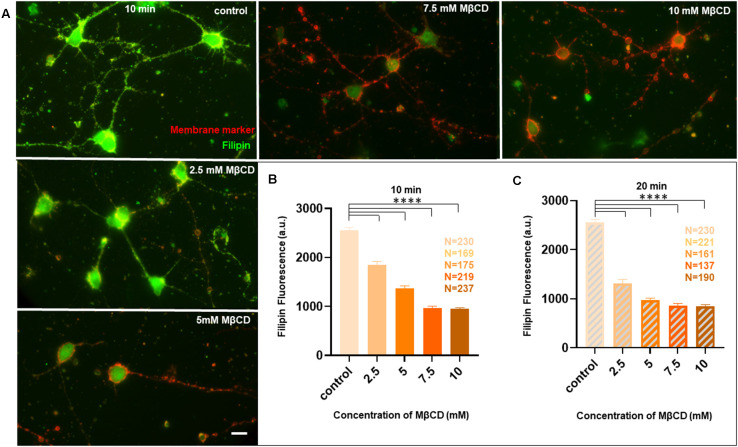
Membrane cholesterol labeling by Filipin III shows altered fluorescence, dependent upon dosage and duration of cholesterol sequestering, consistent with neuro-morphological defects. Neurons at DIV1 were transiently treated with varying concentrations of MβCD for 10 min and 20 min to sequester cholesterol. The cells were labeled with a lipid raft membrane marker (red) and Filipin III (green) and imaged at DIV3. **(A)** A gallery of control and cells treated with varying concentrations of MβCD for 10 min is presented. Both channels in all panels are thresholded with the same values for comparison. **(B)** Represents the absolute fluorescence of Filipin III for cells treated with varying concentrations of MβCD for 10 min. **(C)** Represents the absolute fluorescence of Filipin III for cells treated with varying concentrations of MβCD for 20 min. Fluorescence of Filipin III marks unesterified cholesterol on the membrane. Cholesterol sequestering decreased Filipin III fluorescence, but the effect was severe for 20 min compared to 10 min of cholesterol perturbation, consistent with the corresponding neuro-morphological defects observed. N denotes the number of cells from three independent cultures. One-way Anova was performed to test the significance of the difference between the means. ****p < 0.0001. Scale bar indicates 20 μm.

Since we observed acute neuro-morphological and polarity defects in response to varying levels of cholesterol sequestering, we quantified membrane cholesterol in parallel by an unesterified cholesterol assay by labeling with Filipin III after treatment with different MβCD concentrations (Figure 5). Filipin III is known to be highly photo-unstable; therefore, a lipid raft membrane marker which labels overlapping regions was used as a reference for focusing the cells. We observed that Filipin fluorescence decreased 2.5 to 3 times from the control condition in response to increased sequestering of membrane cholesterol (Figures 5A,B, Table 4). The results were consistent with the dose response curve of Figure 2. We also extended the study to Filipin labeling of cells which had undergone a longer duration of cholesterol sequestering such as 20 min. We observed a similar pattern for alteration in Filipin fluorescence, but a stronger response with a longer duration of perturbation, consistent with the dose response curve of Figure 3 (Figure 5C, Table 4). We also observed a higher membrane damage for the cells undergoing longer cholesterol perturbation (20 min) compared to the short duration (10 min), accounting for the higher variability in Filipin fluorescence in these cells (Table 4).

**Table 4:** Alteration in fluorescence of Filipin III, labeling unesterified membrane cholesterol, upon transient sequestering of cholesterol for varying durations.

Sample	Control	2.5 mM MβCD	5 mM MβCD	7.5 mM MβCD	10 mM MβCD
Filipin fluorescence (10 min)	2556 ± 60.27	1855 ± 65.48	1371 ± 52.07	972 ± 33.04	953.8 ± 19.2
Filipin fluorescence (20 min)	2556 ± 60.27	1311 ± 82.02	969.9 ± 44.85	855.6 ± 51.22.	842.7 ± 39.26

*The table illustrates absolute levels of Filipin III fluorescence in rat hippocampal neurons upon transient cholesterol sequestering for 10 min and 20 min using varying concentrations of the cholesterol sequestering drug MβCD. Since all experiments were performed on the same cultures, a single control is used for both durations of perturbation. Mean ± SEM is presented for N number of cells as shown in Figure 5 from three independent cultures*.

Therefore, we assessed whether the viability was compromised for cells undergoing cholesterol sequestering for longer durations. Cholesterol perturbation was performed at a high concentration (10 mM MβCD) for varying durations namely, 10 min, 20 min, and 30 min (Supplementary Figure 3). The viability assay was performed by two methods. In the first, the membrane damage to the cells induced upon transient cholesterol sequestering at DIV1 for varying durations was assessed at DIV3 in live cells. The control and cholesterol sequestered cells were labeled using a Live/Dead cell imaging kit which marked Calcein AM (green) and Bobo-3 Iodide (orange) as live and dead cell indicators, respectively (Supplementary Figure 3A). The normalized ratio of dead to live cells showed significantly higher levels for 20 min and 30 min compared to 10 min, whose levels were similar to control values (Supplementary Figure 3B, Supplementary Table 1). The assay verified a higher population of cells with membrane integrity compromised with a longer duration of cholesterol perturbation. In the second method, the control and treated cells at DIV3 were fixed and labeled with an antibody recognizing endogenous levels of activated Caspase 3 (orange), and counter labeled with DAPI (Blue, Supplementary Figure 3C). Similar to the live cell assay, the normalized ratio of the percentage of dead vs. live cells showed similar levels between control and 10 min perturbation, in contrast to a significantly higher population of cells entering apoptosis after 20 min and 30 min cholesterol sequestering (Supplementary Figure 3D, Supplementary Table 1). Since our results showed minimal membrane damage and viability issues with short cholesterol sequestering similar to control values, subsequent rescue experiments were performed with the short cholesterol perturbation of 10 min.

### Transient Application of Exogenous Cholesterol Rescues Neurite Outgrowth and Polarity Defects After Cholesterol Perturbation

**Figure 6:**
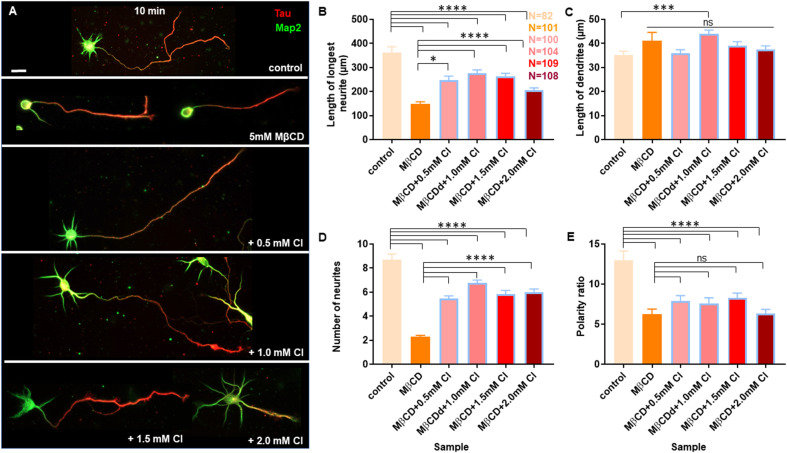
Transient cholesterol replenishment rescues neuronal developmental defects from cholesterol sequestering. Neurons at DIV1 were transiently treated with 5 mM MβCD (referred to as MβCD in the figure) for 10 min to sequester cholesterol. The treated neurons were immediately supplemented with varying concentrations of exogenous cholesterol (Cl) for 10 min. The cells were grown to DIV3 and labeled with Tau (red) and Map2 (green). **(A)** A gallery of control and cells treated with 5 mM MβCD followed by varying concentrations of cholesterol is presented. **(B–E)** Represents the length of the longest neurite **(B)**, length of dendrites **(C)**, number of neurites in each cell **(D)**, and the neuronal polarity ratio **(E)** of the treated cells. The developmental defects observed for cholesterol sequestered neurons were significantly rescued upon supplementing exogenous cholesterol. N denotes the number of cells from three independent cultures. One-way Anova was performed to test the significance of the difference between the means. Significance was tested for each sample against the control as well as against MβCD alone. ****p < 0.0001, ***p < 0.001, *p < 0.05, and ns non-significant. Scale bar indicates 20 μm.

**Figure 7:**
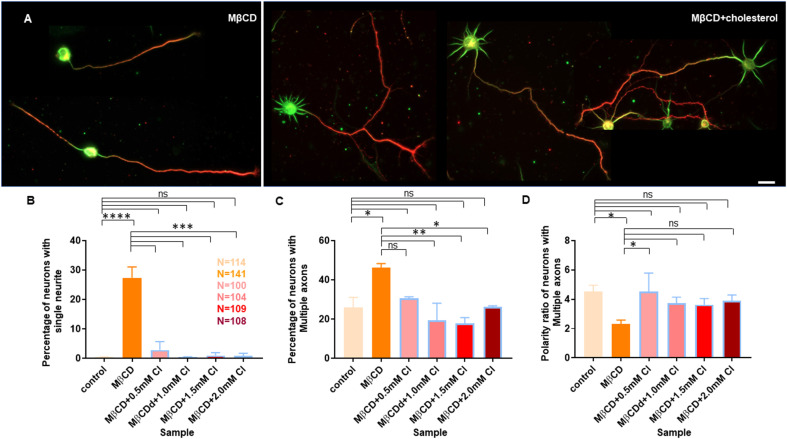
Transient cholesterol replenishment rescues neurite arborization and polarity defects caused by cholesterol sequestering. **(A)** Depicts the morphological transition of neurons upon cholesterol sequestering and transient replenishment with exogenous cholesterol. **(B)** Percentage of cells with single neurites upon transient application of 5 mM MβCD followed by varying amounts of exogenous cholesterol for 10 min. **(C)** Percentage of cells displaying supernumerary axons upon transient application of 5 mM MβCD followed by varying amounts of exogenous cholesterol for 10 min. **(D)** Polarity ratio of neurons exhibiting supernumerary axons as calculated from **(C)**. Neuronal arborization and polarity defects in cholesterol sequestered neurons were significantly rescued upon transient cholesterol replenishment. In the population of neurons displaying supernumerary axons, the polarity ratio was increased to control values in cholesterol supplemented cells, showing their reversal to an asymmetric growth pattern. N denotes the number of cells from three independent cultures. One-way Anova was performed to test the significance of the difference between the means. ****p < 0.0001, ***p < 0.001, **p < 0.01, *p < 0.05. Scale bar indicates 20 μm. ns, non-significant.

Cholesterol sequestering affected axonal and dendritic neurite outgrowth as well as neuronal polarity in a distinct manner. Therefore, we asked whether these developmental defects can be reversed by transient supplementation of membrane cholesterol. This would also address whether the effects we observed upon cholesterol sequestering were specific and direct rather than secondary, since MβCD is also known to affect the cytoskeleton. For this purpose, we treated the hippocampal neuronal cultures at DIV1 with 5 mM MβCD for 10 min, which showed a significant yet moderate effect on neuronal development (Figure 2). The treated cells were washed and immediately supplemented with exogenous cholesterol (Cl) at varying concentrations (0.5 mM, 1.0 mM, 1.5 mM, and 2.0 mM) for 10 min. The cells were washed and grown till DIV3, when they were fixed, immunolabeled with Tau and Map2 and imaged (Supplementary Figure 2Aii). We found that brief cholesterol supplementation of the MβCD treated cells significantly rescued many of the severe developmental defects generated by cholesterol sequestering (Figures 6 and 7).

A gallery of cells including control, MβCD treated (5 mM), and cholesterol supplemented cells is presented (Figures 6A and 7A). On transient cholesterol application, the length of the longest neurite increased from 149.9 ± 7.74 μm to 275.2 ± 14.78 μm, depending on the concentration of the exogenous cholesterol supplemented (Figure 6B, Table 5). Higher cholesterol concentration resulted in retarded axonal length, emphasizing the critical window of the membrane cholesterol content in supporting growth. Cholesterol replenishment, in general, reduced the average length of dendrites of cholesterol sequestered cells to control values (Figure 6C, Table 5). The number of neurites per cell also significantly increased from 2 ± 0.1 in MβCD treated cells to 7 ± 0.2 in cholesterol supplemented cells (Figure 6D, Table 5). The extent of rescue was critically dependent on the amount of replenished cholesterol. Consistently, we observed that the percentage of cells with a single neurite disappeared completely (0 ± 0) or to negligible values from a significant population of 27 ± 4% in MβCD treated cells upon brief cholesterol replenishment (Figure 7B, Table 5). On the contrary, the neuronal polarity ratio displayed a partial rescue upon replenishment (8.25 ± 0.61) when compared to cholesterol sequestered cells (6.22 ± 0.65; Figure 6E, Table 5). Similar to neurite outgrowth, the rescue of polarity was also critically dependent on the replenished cholesterol and behaved similar to cholesterol sequestered cells at high cholesterol concentrations (Figure 6E, Table 5). This strongly supports the critical nature of membrane cholesterol concentration on neuronal polarity. Consistently, we observed that the percentage of cells with supernumerary axons reduced drastically for a small window of replenished cholesterol concentration, emphasizing the critical role of fine membrane cholesterol balance in axo-dendritic specification and neuronal polarity establishment (Figure 7C, Table 5). The neuronal polarity ratio of the cholesterol replenished cells with multiple axons (4.51 ± 1.27) was higher than that of cholesterol sequestered cells (2.29 ± 0.29) and similar to that of control cells (4.52 ± 0.44), thereby verifying that the asymmetric nature of neurite growth had returned on retaining the membrane cholesterol balance (Figure 7D, Table 5). The results confirmed the critical nature of membrane cholesterol content in neurite outgrowth and neuronal polarity establishment.

**Table 5:** Rescue of developmental defects of cholesterol sequestered neurons by transient application of exogenous cholesterol.

Sample	Control	MβCD	MβCD + 0.5 mM Cl	MβCD + 1.0 mM Cl	MβCD + 1.5 mM Cl	MβCD + 2.0 mM Cl
Length of the longest neurite (μm)	361.9 ± 24.58	149.9 ± 7.74	246.5 ± 17.83	275.2 ± 14.78	262.9 ± 12.59	205.1 ± 10.29
Length of dendrites (μm)	35.23 ± 1.54	41.07 ± 3.53	35.93 ± 1.46	44.03 ± 1.55	38.98 ± 1.76	37.54 ± 1.46
Number of neurites per cell	9 ± 0.5	2 ± 0.1	6 ± 0.2	7 ± 0.2	6 ± 0.3	6 ± 0.3
Polarity ratio	12.94 ± 1.18	6.22 ± 0.65	7.87 ± 0.69	7.60 ± 0.69	8.25 ± 0.61	6.33 ± 0.51
Percentage of cells with single neurite (%)	0 ± 0*	27 ± 4	3 ± 2	0 ± 0*	1 ± 1	1 ± 1
Percentage of cells with multiple axon (%)	26 ± 5	46 ± 2	31 ± 0.8	19 ± 9	18 ± 3	26 ± 0.6
Polarity ratio of cells with multiple axon	4.52 ± 0.44	2.29 ± 0.29	4.51 ± 1.27	3.73 ± 0.41	3.63 ± 0.42	3.89 ± 0.41

The neuro-morphological perturbations induced by transient cholesterol sequestering were rescued upon transient application of exogenous cholesterol. The table compares the alterations in the morphological properties of control, cholesterol sequestered, and cholesterol supplemented neurons. MβCD refers to 5 mM MβCD. *0 values have been replaced with negligible values in Figure 7B for the sake of display. Mean ± SEM is presented for N number of cells as shown in Figures 6 and 7 from three independent cultures.

### Increasing Cholesterol Content on the Cell Membrane Alters Growth but Not Polarity

**Figure 8:**
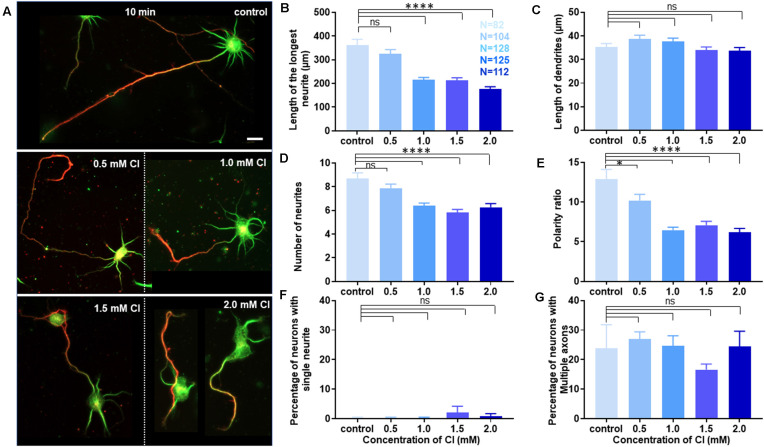
Membrane cholesterol enhancement alters growth but does not shift polarity. **(A)** A gallery of control and cells transiently treated with varying concentrations of cholesterol (Cl) is presented. Higher cholesterol concentrations altered neuronal architecture and morphology, but also generated cells with axons growing and curling back over the soma. **(B–E)** Represents the length of the longest neurite **(B)**, length of dendrites **(C)**, number of neurites in each cell **(D)**, and the neuronal polarity ratio **(E)** of the treated cells. **(F,G)** Represents the percentage of treated neurons displaying single neurites **(F)** and supernumerary axons **(G)**, respectively. Transient membrane cholesterol enhancement significantly perturbed axonal growth and neurite arborization but did not hamper dendritic growth. The neuronal polarity ratio shifted to lower values upon cholesterol enhancement, denoting symmetric growth. Interestingly, there was no significant population of treated neurons with single neurites or displaying multiple axons, confirming that cholesterol enhancement complemented dendritic extension, but not polarity shift to develop supernumerary axons. N denotes the number of cells from three independent cultures. One-way Anova was performed to test the significance of the difference between the means. ****p < 0.0001, *p < 0.05, and ns non-significant. Scale bar indicates 20 μm.

Neuronal developmental defects induced by cholesterol sequestering were rescued by the transient application of exogenous cholesterol. Therefore, we wanted to address the effect of increasing the membrane cholesterol concentration on neuronal development. The rat hippocampal neurons at DIV1 were transiently treated with varying concentrations of exogenous cholesterol (0.5 mM, 1.0 mM, 1.5 mM, and 2.0 mM) for 10 min (Supplementary Figure 2Aiii). The cells were washed and grown till DIV3, when they were fixed, immunolabeled with axonal and dendritic markers, and imaged. A gallery of cells treated transiently with varying concentrations of cholesterol is presented in Figure 8A. We observed that the normal neuronal morphology was retained for a short concentration window of supplemented cholesterol and was lost at high cholesterol concentrations, emphasizing the relevance of the correct membrane lipid balance for the normal neuronal architecture. Cholesterol imbalance altered the shape of the soma and resulted in axons growing and curling back towards the soma instead of leading away from it, increasing the probability of generating ectopic synapses (Figure 8A). While supplementing extrinsic cholesterol rescued neurite outgrowth of cholesterol perturbed cells, cholesterol addition to the normal neurons resulted in attenuated growth. This attenuation was significant and augmented with increasing cholesterol concentration. The length of the longest neurite reduced from 361.9 ± 24.58 μm in control cells to 177.1 ± 9.06 μm in cells treated with higher amounts of cholesterol (Figure 8B, Table 6). In contrast to cholesterol sequestering which affected axon and dendrites in an opposing manner, cholesterol enhancement did not significantly alter the growth of dendrites (Figure 8C, Table 6). However, cholesterol enhancement negatively affected neurite outgrowth and neuronal arborization and reduced the average number of neurites per cell from 9 ± 0.5 in control cells to 6 ± 0.3 in treated cells (Figure 8D, Table 6). Contrary to cholesterol sequestering, cholesterol enhancement did not generate a significant population of neurons with a single neurite (Figure 8F, Table 6). The most significant effect of cholesterol enhancement was observed for the neuronal polarity ratio which sharply decreased from 12.94 ± 1.18 to 6.18 ± 0.5 upon increasing the membrane cholesterol concentration (Figure 8E, Table 6). The decrease in polarity ratio indicated the reversal of neurons to symmetric growth following cholesterol enhancement. Interestingly, contrary to cholesterol sequestering, cholesterol enhancement did not significantly shift polarity, though it altered neurite outgrowth and extension. Consistently, the percentage of cells displaying supernumerary axons was not significantly altered upon cholesterol enhancement, when compared to control neurons (Figure 8G, Table 6). These results showed that membrane cholesterol enhancement altered neurite outgrowth but not polarity. Together these observations support the critical nature of membrane cholesterol balance in regulating neurite outgrowth and polarity, and in maintaining the normal neuronal morphology and architecture.

**Table 6:** Developmental alterations of rat hippocampal neurons upon transient application of exogenous cholesterol for 10 min.

Sample	Control	0.5 mM Cl	1.0 mM Cl	1.5 mM Cl	2.0 mM Cl
Length of the longest neurite (μm)	361.9 ± 24.58	324.3 ± 18.19	215.4 ± 9.63	212.3 ± 11.57	177.1 ± 9.06
Length of dendrites (μm)	35.23 ± 1.5	38.72 ± 1.6	37.66 ± 1.41	33.99 ± 1.34	33.63 ± 1.44
Number of neurites per cell	9 ± 0.5	8 ± 0.3	6 ± 0.2	6 ± 0.3	6 ± 0.3
Polarity ratio	12.94 ± 1.18	10.19 ± 0.79	6.46 ± 0.37	7.08 ± 0.49	6.18 ± 0.5
Percentage of cells with single neurite (%)	0 ± 0*	0 ± 0*	0 ± 0*	2 ± 2	1 ± 1
Percentage of cells with multiple axon (%)	24 ± 8	27 ± 2	25 ± 3	17 ± 2	25 ± 5

The table illustrates morphological alterations of neurons transiently treated with varying concentrations of exogenous cholesterol. *0 values have been replaced with negligible values in Figure 8F for the sake of display. Mean ± SEM is presented for N number of cells as shown in Figure 8 from three independent cultures.

## Discussion

Molecular mechanisms underlying neuronal polarity have been investigated for decades. However, our understanding of the complex interplay among various signaling pathways in regulating neurite outgrowth and polarity establishment remains poorly understood. The complexity of the fundamental mechanisms that regulate axonal and dendritic specification makes it challenging, yet interesting. This study addresses the role of one of the key players of this network, namely membrane cholesterol which has been shown to be a crucial player in orchestrating multiple processes and pathways. Alterations in membrane cholesterol homeostasis have been correlated with different pathophysiological conditions including Alzheimer’s disease, Parkinson’s disease, Huntington’s disease, and Niemann Pick type C disease (Vance, 2012; Wang and Song, 2012; Leoni and Caccia, 2015; Hussain et al., 2019). Recent evidence has linked key proteins involved in these disease conditions to the regulation of lipid and cholesterol homeostasis, further underscoring the consequences of mis-regulation of these pathways and disease pathology (Pierrot et al., 2013; Grimm et al., 2017; Cho et al., 2020). Cholesterol has also been linked to modification and maturation of signaling molecules such as hedgehog proteins, critically involved in embryogenesis (Porter et al., 1996; Blassberg and Jacob, 2017). In addition to neurodegenerative conditions, disorder in cholesterol synthesis has been reported to cause multiple human malformation syndromes including Smith-Lemli-Opitz syndrome (SLOS; Porter and Herman, 2011). Reports show altered signaling of multiple GTPases impairing normal axonal and dendritic growth, potentially in a cholesterol dependent manner, contributing to the neurocognitive deficits found in SLOS (Jiang et al., 2010). All this evidence emphasizes the requisite for a better understanding of the critical regulation of membrane cholesterol during different phases of development, particularly during neurite outgrowth and polarity establishment, the mis-regulation of which can lead to multiple pathophysiological conditions.

Primary cultures of rodent hippocampal neurons are a widely used and amenable system to address molecular mechanisms underlying neuronal growth and polarity. The development of hippocampal neurons in culture has been classified into different stages (Stage 1–5): development of protrusions or lamellipodia (stage 1), neurite outgrowth (stage 2), symmetry breaking and axonal extension (stage 3), dendritic growth (stage 4), and formation and maturation of synapses (stage 5; Dotti et al., 1988). Most of the studies on the effects of cholesterol alterations on neurite outgrowth have been primarily addressed in stages 3–5, when neuronal polarity has already been established (Fan et al., 2002; Ko et al., 2005). In this study, we undertook a detailed biophysical characterization of the neuro-morphological effects after altering the membrane cholesterol in a controlled and minimally invasive manner transiently, so as not to disturb its natural growing environment. Due to the challenging nature of this early developmental stage to determine polarity establishment, in addition to qualitative polarity analysis using conventional axo-dendritic markers, different quantitative parameters were also considered including the neuronal polarity ratio, which is the ratio of the longest neurite to the average length of the dendrites. This gives a reliable readout of the polarity status of a neuron, consistent with the morphological and molecular signatures during neuronal development. The protocols were optimized to generate a homogenous neuronal population, and the different quantitative parameters for assessing neuronal development and polarity were thoroughly assessed for their variability between culturing conditions. Experiments were performed in the same sets of neuronal cultures to minimize the variability between cultures. The robust approach of this study allowed us to decipher multiple components of neuronal development including neurite outgrowth and polarity establishment in a reliable manner.

We observed that transient cholesterol sequestering, irrespective of the duration of perturbation, affected axons and dendrites in a distinct manner, attenuating axonal growth and promoting dendritic growth. This manipulation generated two subsets of neuronal populations, those with a single neurite or with multiple axons. Though both cholesterol sequestering as well as cholesterol enhancement affected neurite outgrowth, only the former shifted neuronal polarity. Transient cholesterol replenishment of sequestered neurons rescued developmental and polarity defects to a significant extent. However, the rescue was critically dependent on the amount of replenished cholesterol, emphasizing the importance of precise membrane lipid balance for optimal neurite outgrowth and neuronal architecture.

Previous studies have reported that cholesterol modulation alters neurite outgrowth in a region-dependent manner in the brain. In one study, cholesterol perturbation promoted neurite outgrowth in the hippocampus, but retarded growth in the neocortex (Ko et al., 2005). Since these reported experiments were performed at later stages (3–5), the effects of the perturbations might be dependent on the developmental stage. On the contrary, the increase in the population of neurons displaying multiple axons following cholesterol sequestering, as we have observed, might also explain the differences in the observations. Membrane cholesterol deficiency has been reported to affect Tau phosphorylation and microtubule depolymerization in axons. It also causes selective inhibition of dendritic outgrowth by affecting MAP2 phosphorylation and microtubule destabilization, consistent with our observation of an increase in the population of neurons with a single neurite on cholesterol sequestering (Fan et al., 2001, 2002). Neuronal cholesterol metabolism has been shown to modulate dendritic arborization and synaptic content, emphasizing its importance in mature neurons as well (Moutinho et al., 2016). Cholesterol is a critical player in defining membrane fluidity, underscoring its key role in regulating the lateral diffusion of molecules involved in different signaling pathways, including polarity establishment, synaptic transmission, and pathophysiological conditions. Previous studies have shown that the lateral diffusion and membrane nano-organization of many of these key molecules is defined by several factors including intracellular ionic levels, genetic alterations or the membrane potential of the cell (Nair et al., 2013, 2020; Kedia et al., 2020a, b; Tanwar et al., 2021). These factors could provide potential feedback to the membrane lipid compartments for defining the protein-lipid compartmentalization to regulate these pathways.

The cholesterol sequestering drug, Methyl β cyclodextrin, has been shown to act as an actin depolymerizing agent as well (Mundhara et al., 2019). The cytoskeletal modifications on cholesterol perturbation by MβCD were similar to that of Latrunculin B, another actin depolymerizing drug (Mundhara et al., 2019). Actin depolymerization at low concentrations has been shown to promote neurite outgrowth and extension in hippocampal neurons, whereas it was inhibited at high concentrations (Chia et al., 2016). Since developmental defects observed by cholesterol sequestration by MβCD might involve effects from both lipid and cytoskeletal modifications, it was important to dissect out the influence of cholesterol alteration vs. cytoskeletal rearrangement on the developmental outcome. Using an unesterified cholesterol assay, we confirmed that the membrane cholesterol content was effectively reduced by transient cholesterol sequestering, and that the reduction was dependent upon the dosage of MβCD. Also, we observed a stronger reduction in membrane cholesterol levels on increasing the duration of perturbation, consistent with our observations of severe neuro-morphological defects associated with longer cholesterol sequestering. Our transient cholesterol replenishment studies significantly rescued the neuronal developmental defects observed on sequestering, clearly emphasizing a critical role for membrane cholesterol in determining the normal neuronal architecture and polarity.

Interestingly, a significant population of supernumerary axons was observed on cholesterol depletion, which augmented with increasing concentrations of MβCD. Actin depolymerization in developing neurons (stage 2) has been shown to promote multiple axons (Bradke and Dotti, 1999). However, since this population was significantly reduced to normal levels on cholesterol supplementation, a critical role for cholesterol in determining neuronal polarity was concluded, though the overlap of cytoskeletal modifications cannot be overruled. Cholesterol depletion has been shown to alter ion channels (Amsalem et al., 2018) and surface receptor signaling (Fukui et al., 2015; Antonini et al., 2018), as well as lipid raft integrity (Rosello-Busquets et al., 2019). Recent studies emphasize the critical role of the Rap GTPase regulator Sema3A and its receptor Plexin-A1 in suppressing supernumerary axons in hippocampal neurons (Wang et al., 2018). Hippocampal neurons from Sema3A knockout mice displayed multiple axons (Wang et al., 2018). Earlier studies have also shown the involvement of Sema3C in inducing lipid raft dependent endocytosis (Salikhova et al., 2008). It remains to be seen whether cholesterol sequestering alters the membrane binding of Sema3A, which is thought to act as an autocrine signal for the establishment of neuronal polarity. The binding and activation by Sema3A might also be lipid raft dependent processes, which would be compromised by cholesterol depletion. This could in turn leave the Plexin-A type containing receptors in their autoinhibited state, rendering the generation of multiple axons by increased Rap1B activity (Wang et al., 2018).

Cellular toxicity for Methyl β Cyclodextrin has been reported previously (Peake and Vance, 2012; Rosello-Busquets et al., 2019). Our studies also show acute membrane damage and compromised viability with a longer duration of incubation, particularly at higher concentrations. Cholesterol depleting enzymes such as ChOx and statins (Nystatin, Lovastatin) alone or in combination with small molecules to inhibit cholesterol biosynthesis might be alternate choices for studying the influence of membrane cholesterol at early developmental stages (Simons et al., 1998; Rosello-Busquets et al., 2019). However, the effect of dosage and duration of these drugs would need to be optimized as well as toxicity assessed according to the developmental stage of the cells since the outcome might vary.

Emerging evidence suggests cholesterol and lipid rafts to be potential therapeutic targets to address different neurological disorders (Vance, 2012; Vona et al., 2021). Different approaches for regulating cholesterol homeostasis have yielded promising results in addressing neurological disorders such as NPC disease (Peake and Vance, 2012). Although neurons have an autonomous cholesterol synthesis machinery, the majority of their cholesterol need is provided by glial cells—microglia in young neurons and, astrocytes and oligodendrocytes through the myelin sheath in the adult brain (Funfschilling et al., 2012). The metabolic support from the glial cells might be critical either as an extrinsic supply for maintaining the neuronal membrane lipid balance or for triggering the signaling pathways regulating the neuronal intrinsic cholesterol supply. Thus, the neuron-glia exchange of cholesterol might have a strong implication on the polarity establishment and maintenance of the neuronal architecture. Our results emphasize the implications of a transient lipid imbalance on neuronal architecture as well as on neural circuitry. Our findings also show that it is possible to reverse the neuronal developmental defects caused by cholesterol deficiency by modulating membrane cholesterol during the early developmental stages. Together, regulating cholesterol levels is a potential therapeutic platform for addressing many of the neurodegenerative and developmental disorders that depend on cholesterol homeostasis. Though previous studies using small molecules such as cyclodextrin to regulate membrane cholesterol have been promising, their applications have been limited by the toxicity they induce (Peake and Vance, 2012). Therefore, genetic modulation of glial cholesterol exporters to modify the neuronal membrane lipid integrity and cholesterol homeostasis might be key to address many of the disorders depending on cholesterol homeostasis in the brain (Karasinska et al., 2009; Courtney and Landreth, 2016). Multiple studies show altering the overall glial content in the brain as a therapeutic strategy to ameliorate neurotoxicity and neuropathology, indicating the importance of global cholesterol homeostasis in the progression of neurodegenerative conditions such as AD (Asai et al., 2015; Spangenberg et al., 2019). However, the relevance of the glial metabolic support on neuronal development in these diseases still needs to be addressed. Transgenic animals labeling different neuronal subtypes and single gene editing techniques including Crispr-Cas9 provide feasible choices for sparse labeling of neurons and quantification of novel parameters such as the neuronal polarity ratio to study the polarity defects *in vivo* (Young et al., 2008; Ran et al., 2013).

In conclusion, mis-regulation of cholesterol metabolism and membrane lipid homeostasis has been correlated with several neurological disorders. This study addresses the fundamental biophysical aspects of cholesterol regulation on membrane physiology and development using rat hippocampal neurons as a model system. Because of the technical challenges in studying polarity establishment during early neuronal development, most of the previous research in this area has been conducted in later stages of development when neuronal polarity has already been established. This is one of the very few studies which has addressed the role of cholesterol homeostasis in neuronal development and differentiation at the very early stages of neurite growth. Our study signifies the stark effect of a transient alteration in membrane cholesterol balance on neurite outgrowth and polarity establishment. It also emphasizes the significance of addressing this condition at an early developmental phase, where the developmental defects can be rescued to a significant extent by modulating the lipid membrane balance in a precise manner. We observed distinct effects for transient cholesterol sequestering on the development of axons and dendrites and on neuronal polarity establishment. Differential effects were also observed for cholesterol enhancement and sequestering on neurite outgrowth and polarity. Thus, this study identifies membrane cholesterol as a critical factor for axo-dendritic specification and a key molecule for determining neuronal architecture. It also provides insights into regulating membrane cholesterol levels as a potential therapeutic target to address neurodegenerative and developmental disorders that depend on cholesterol homeostasis.

## Data Availability Statement

The original contributions presented in the study are included in the article/Supplementary Material, further inquiries can be directed to the corresponding author.

## Ethics Statement

All animal procedures to prepare primary cultures were performed with approval and clearance from Animal Ethics Committee, Indian Institute of Science.

## Author Contributions

The project was conceptualized and executed by MJ. All experiments including cell culturing, drug treatments and labeling were done by MJ. Microscopy imaging was done by MJ and CC. Data analyses were done by MJ, AS, and CC. The manuscript was written by MJ. All authors contributed to the article and approved the submitted version.

## Conflict of Interest

The authors declare that the research was conducted in the absence of any commercial or financial relationships that could be construed as a potential conflict of interest.

## Publisher’s Note

All claims expressed in this article are solely those of the authors and do not necessarily represent those of their affiliated organizations, or those of the publisher, the editors and the reviewers. Any product that may be evaluated in this article, or claim that may be made by its manufacturer, is not guaranteed or endorsed by the publisher.
