# Supplementary material for: Membrane Cholesterol Is a Critical Determinant for Hippocampal Neuronal Polarity
Source: Front Mol Neurosci. 2021 Oct 21;14:746211. doi: 10.3389/fnmol.2021.746211 (PMC8566733; doi:10.3389/fnmol.2021.746211)
Supplement: Supplementary file 1 [file Data_Sheet_1.pdf]

Supplementary Material

1 Supplementary Figures and Tables

1.1 Supplementary Figures

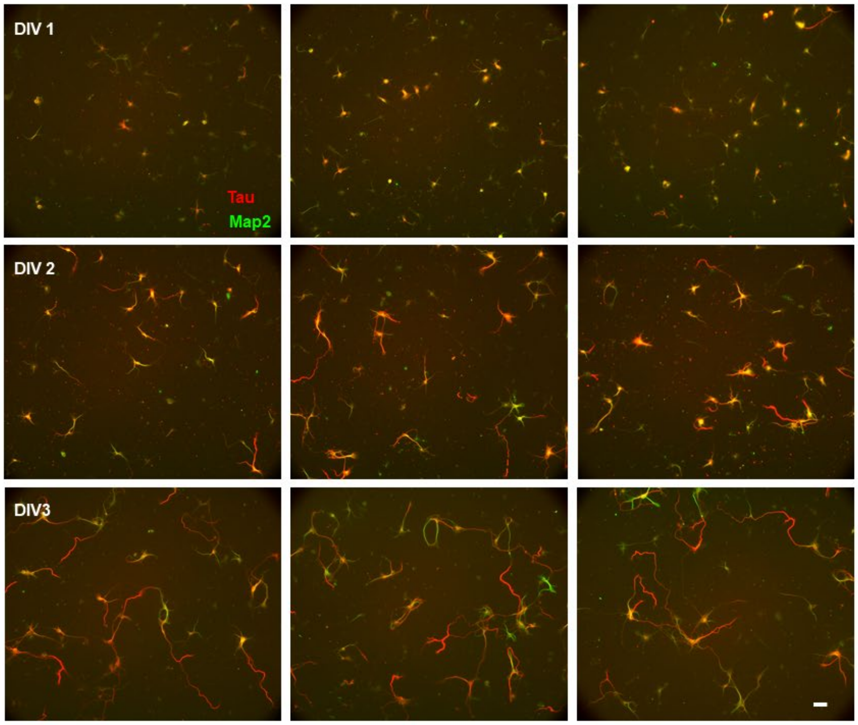

Supplementary Figure 1 Developmental profile of primary cultures of rat hippocampal neurons. Neurons were grown to three developmental stages namely DIV 1, 2 and 3, fixed and labelled with Tau (red) and Map2 (green) as axonal and dendritic markers, respectively. A gallery of cells from DIV 1 (top), DIV 2 (center) and DIV 3 (bottom) is presented. Images were acquired using a low magnification objective. Both channels in all panels are thresholded with same values for comparison. Images represent the majority of cells undifferentiated at DIV 1 and differentiated at DIV 3. Scale bar indicates 40 µm.

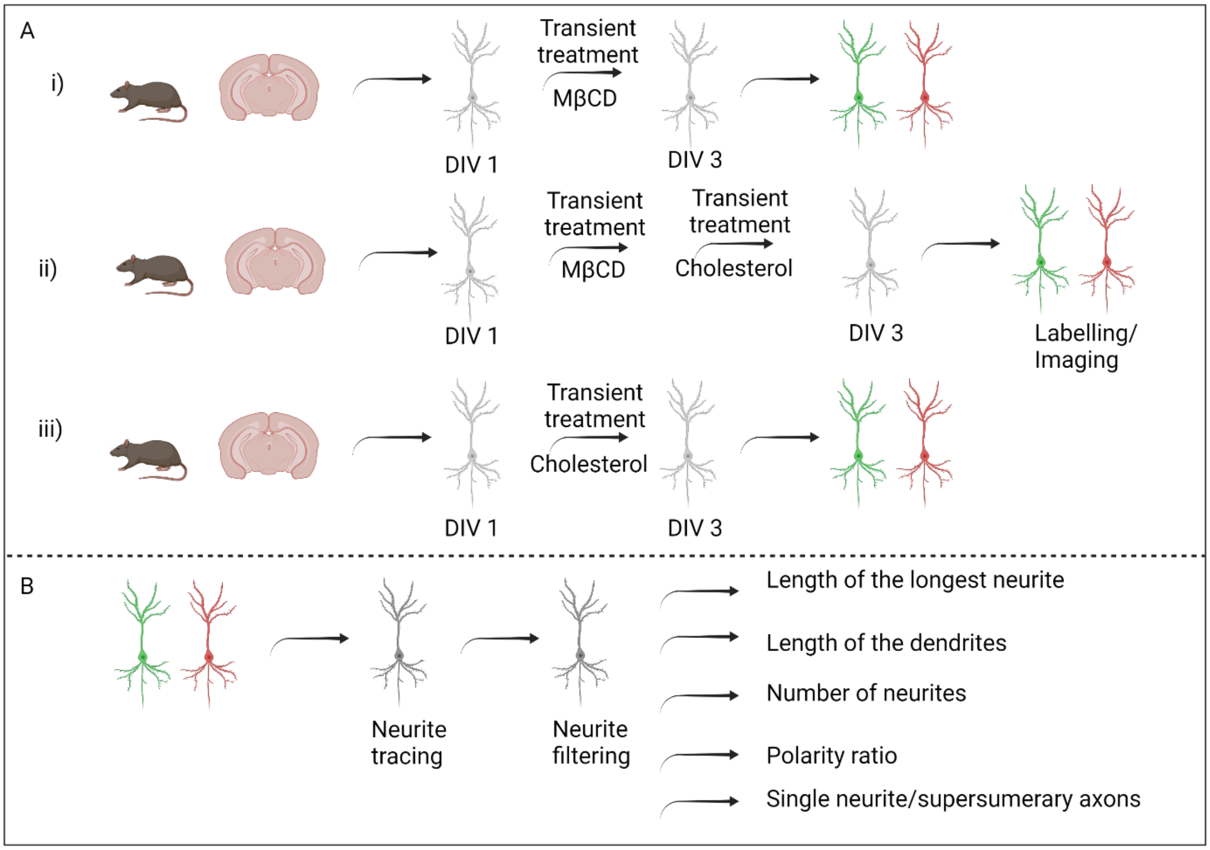

Supplementary Figure 2 Schematics of experimental and analytical procedures. **A)** i) Rat hippocampal neurons at DIV1 were transiently treated with MβCD alone at varying concentrations (2.5 mM-10 mM) for different durations (10/20 min), ii) or followed by brief replenishment with varying concentrations of cholesterol (0.5 - 2 mM, 10 min), iii) or transiently treated with varying concentrations of cholesterol alone (0.5 - 2 mM, 10 min). Transiently treated cells were washed and grown to DIV3, where they were fixed, immunolabelled for axonal and dendritic markers and imaged. **B)** Neurite tracing was performed manually on maximum intensity projections of 3D stacks of the acquired images. Neurite filtering was done to exclude filopodia. Neurites >10 µm were selected for the rest of the quantitation including length of the longest neurite, length of dendrites, number of neurites per cell and polarity ratio. Polarity ratio was calculated from the ratio of the longest neurite to the average length of dendrites from each cell. Number of axons was quantified based on the number of Tau positive neurites. The image was created using Biorender.com.

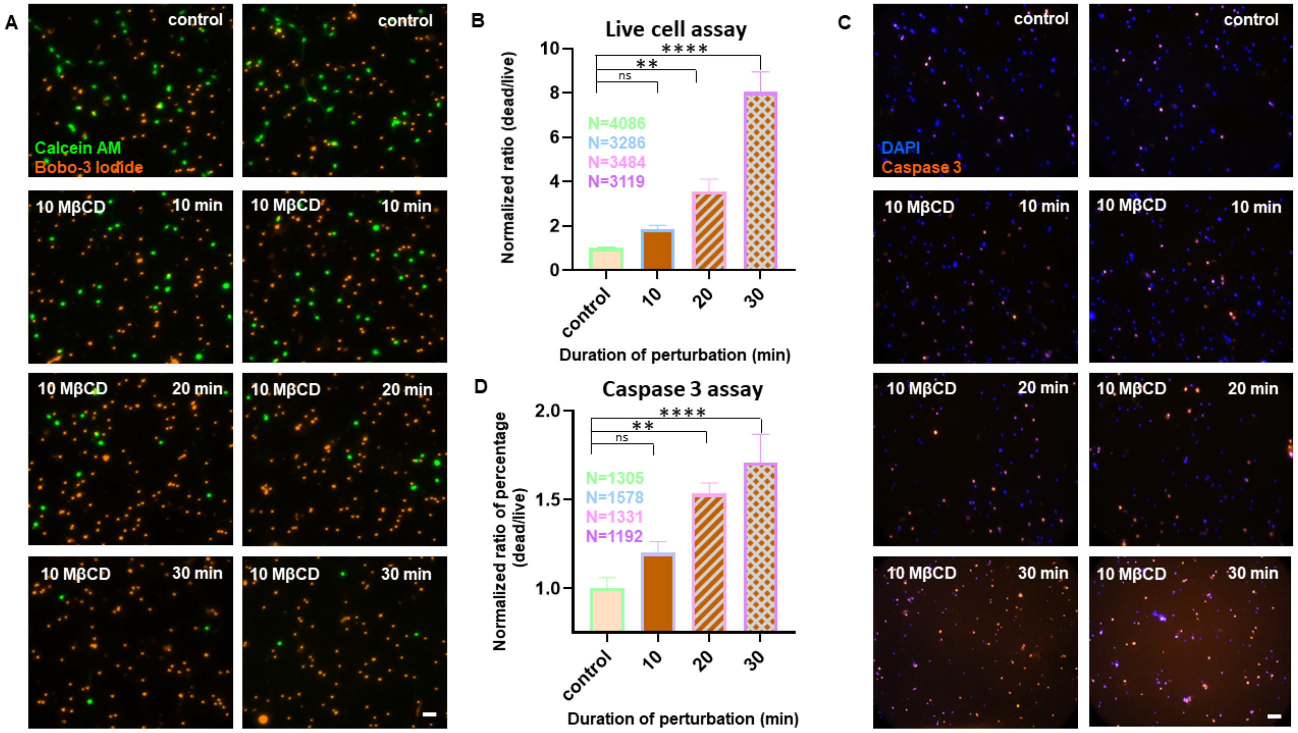

Supplementary Figure 3 Cell viability assays confirm higher population of cells entering apoptosis upon transient sequestering of cholesterol for longer durations. **A-D)** Neurons at DIV1 were transiently treated with 10mM MβCD for various durations, namely 10 min, 20 min and 30 min to sequester cholesterol. **A)** Control and treated cells at DIV3 were live labelled using a Live/Dead cell imaging kit. **B)** The ratio of dead (orange) to live (green) cells were quantified, normalized using the mean value of control and plotted for varying durations of cholesterol perturbation. N represents the total number of cells from three independent cultures. **C)** Control and treated cells at DIV3 were fixed and labelled using an antibody marking activated Caspase 3 (orange, Alexa 555) and counter labelled with DAPI (blue). **D)** The ratio of percentage of dead to live cells was quantified, normalized using the mean value of control and plotted for varying durations of cholesterol perturbation. Since all experiments were performed on the same cultures, a single control was used for each assay for different durations of perturbation. Cell viability was compromised with longer duration of cholesterol sequestering. N represents the total number of cells from two independent cultures. One-way Anova was performed to test the significance of the difference between the means. ****indicates $p<0.0001$, ** $p<0.01$ and ns non-significant. Scale bar indicates 40 μm.

1.2 Supplementary Tables

Supplementary Table 1 Cell viability assays upon transient sequestering of cholesterol for varying durations. The table illustrates the ratio of dead to live cells by two different assays (Live cell assay and Caspase 3 assay) upon transient cholesterol sequestering for varying durations using 10mM MβCD. Mean ± S.E.M. is presented for N number of cells as shown in **Supplementary Figure 3** from three and two independent cultures. Since all experiments were performed on the same cultures, a single control is used for each assay for different durations of perturbation. Cell viability was compromised with longer duration of cholesterol sequestering.

**Assay**	**Parameter**	**Control**	**Duration of sequestering by 10mM MβCD (min)**		
			**10**	**20**	**30**
Live cell	Normalized ratio (dead/live)	1±0.04	1.86±0.17	3.57±0.54	8.04±0.9
Caspase 3	Normalized ratio of percentage (dead/live)	1±0.06	1.2±0.06	1.5±0.06	1.7±0.16
